# Colored Reflective Paints with Doped ZnO Fillers for High Cooling Capabilities

**DOI:** 10.3390/nano16181155

**Published:** 2026-09-15

**Authors:** Shaojun Xu, Yan Peng, Zhongqiu Tong, Xingang Li, Feiyan Long, Jie Yu, Shaoyuan Li, Yueming Wang, Wenhui Ma

**Affiliations:** 1 Faculty of Metallurgical and Energy Engineering, Kunming University of Science and Technology, Kunming 650093, China; 2 Jiangxi Construction Engineering Co., Ltd., Nanchang 330000, China; 3 School of Engineering, Yunnan University, Kunming 650091, China

**Keywords:** reflective paints, building cooling energy saving, coloration, ZnO fillers, metal ion-doping

## Abstract

Colored reflective paints are attractive for building cooling and aesthetics. However, coloration of the paints could paradoxically increase the solar absorption, decreasing the cooling capabilities. In this work, we introduced a novel coloration strategy by using metal ion-doped ZnO nanoparticles as fillers to achieve chromatic paints with high cooling capabilities. With inorganic binder of aqueous K_2_SiO_3_ solution, it observed that the Mg-doping enabled the paint to deliver a high cooling power of 103.8 W/m^2^, while Fe-, Cu- and Co-doping strategies created pale buff (L* = 84.6, a* = 2.9, b* = 16.3), olive green (L* = 66.8, a* = −7.9, b* = 18.7), and pale gray (L* = 81.5, a* = −0.6, b* = 2.1) colors with highly remained cooling power of 83.9, 75.4 and 61.1 W/m^2^, respectively. Physical characterizations indicated that the maintained crystal structures and similar band gaps could be the main reasons for high cooling capabilities of the colored paints. In addition, the high temperature differences and strong adhesion capabilities of paints on concrete substrates enabled them to present promising cooling energy savings for buildings.

## 1. Introduction

Along with the worldwide temperature increase, the significantly increased active building cooling energy not only unfortunately further deteriorates the global warming but also increases household expenses for living and manufacturing [1,2]. Employment of reflective paints is an attractive strategy to solve the above-mentioned problems because of their passive cooling capabilities derived from the reflection of incident solar photons and emission of mid-infrared energies [3,4]. Up to now, various white reflective paints have been developed, and some paints have been employed on practical buildings to get promising annual cooling energy-saving performance [5,6,7,8,9]. For instance, a white paint formed by mixed TiO_2_ and SiO_2_ fillers with polystyrene emulsion demonstrated an attractive annual cooling saving capacity of 244.2 kWh m^−2^ in Hong Kong tested on metal container offices [10].

These desirable examples proved the promising applications of the reflective paints. However, there is still a huge obstacle preventing further promotion of its practical application; that is, colored paints are more desirable for the aesthetics and comfort of urban architectures [11,12,13]. White reflective paints can indeed significantly decrease the solar absorption, but they undeniably lead to light pollution, including glaring and night skyglow [14,15,16]. The widely developed strategies to color paints include employment of dyes for pigmentary color [17,18,19] and optical interference for structural color [20,21,22]. Typically, organic, inorganic and fluorescent dyes (such as red-toned perylene [23], Fe_2_O_3_ [24] and CaAlSiN_3_:Eu [25]) can make the paints more vivid but they also result in strong solar light absorption. The realization of structural color is usually based on phenomena of the periodic structures in the paint coatings (such as three-dimensionally ordered macro pores [26]) and the optical interactions between the paint coating and substrate (such as Fabry–Pérot effect [27]), which show advantages of negligible effects on the solar energy absorption but unfortunately lead to drawbacks of high costs and do not ease the large-scale fabrication of buildings. Thus, the development of colored reflective cooling paints simultaneously capturing characters of high cooling capabilities and ease for large-scale fabrication is attractive and still challenging.

In this work, we introduced a new coloration strategy on reflective paints via using metal ion-doping of ZnO fillers with high refractive indexes and large energy gaps. Theoretically, for metal oxides with the above-mentioned two characters, small metal ion doping levels cannot significantly change their wavelength-dependent refractive indexes and extinction coefficients [28,29,30,31], leading the oxides to be colored and still demonstrate low solar absorption behaviors [32,33,34]. With an inorganic binder of aqueous K_2_SiO_3_ solution and by systematically studying the physical characters of low-level (0.5 at%) metal ion-doped ZnO-based paint coatings, it was observed that the Mg-doping slightly increased the energy gap to 3.47 eV, leading to an attractive cooling power of 103.8 W m^−2^. While, Fe-, Cu- and Co-doping methods leaded to colors of pale buff (L* = 84.6, a* = 2.9, b* = 16.3), olive green (L* = 66.8, a* = −7.9, b* = 18.7), and pale gray (L* = 81.5, a* = −0.6, b* = 2.1) with band gaps of 3.24, 3.25 and 3.27 eV, generating desirable cooling powers of 83.9, 75.4 and 61.1 W m^−2^, respectively. In addition, the developed paints can be tightly adhered to various substrates, including wood, rough steel and concrete, indicating their desirable potential for practical applications.

## 2. Experimental Section

### 2.1. Materials

Hexagonal ZnO powder (diameter of ~300 nm, purity of 99.9%), Mg(NO_3_)_2_·6H_2_O (purity of 99.9%), La(NO_3_)_3_·6H_2_O (purity of 99%), Fe(NO_3_)_3_·9H_2_O (purity of 99.9%), Cu(NO_3_)_2_·3H_2_O (purity of 99.9%), and Co(NO_3_)_2_·6H_2_O (purity of 99.9%) were purchased from MACKLIN Company, Shanghai, China. An aqueous K_2_SiO_3_ solution with a modulus of 2.2 was purchased from Aladdin Company, Shanghai, China.

### 2.2. Fabrication of Doped ZnO Powders and Corresponding Paints

The mixture of ZnO and metal nitrate was thoroughly blended in a ball mill using ethanol as the milling medium. The target doping concentration of metal ions was 0.5 at%, and inductively coupled plasma (ICP) tests confirmed that the actual doping level was around this value. After the aqueous mixtures were dried in air at 80 °C, they were transferred into a muffle furnace and annealed at 600 °C for 4 h to get metal ion-doped ZnO powders.

ZnO powder, aqueous K_2_SiO_3_ solution and deionized water were mixed thoroughly in a ball milling machine to get a homogenous paint emulsion. The weight ratio of ZnO powder, aqueous K_2_SiO_3_ solution and deionized water was set as 1:0.8:0.4. The prepared paint was sprayed on various substrates under high-pressure air flow and dried in air at room temperature for 10 days before testing. Wood, rough steel and concrete were used as substrates.

### 2.3. Physical and Radiative Cooling Characterizations

The morphology, crystal structure and chemical bonds of the samples were detected by a scanning electron microscopy (SEM, SIGMA 300, ZEISS, Oberkochen, Germany), an X-ray diffraction (XRD, PANalytical, X’Pert3 Powder, Almelo, the Netherlands) with Cu Kα radiation (λ = 0.15418 nm) and Raman spectroscopy (HORIBA JY LabRA, Kyoto, Japan). The solar reflectance was measured with a Perkin Elmer Lambda 950 UV–Vis–NIR spectrometer with an integrating sphere and a BaSO_4_ optical reference panel. The IR emissivity and FTIR spectra were characterized by a Nicolet iS50 FTIR with a PIKE Technology integrating sphere.

Radiative cooling power was simulated using COMSOL Multiphysics (v5.4). An optical-thermal coupled model with solar irradiance and atmospheric transmittance spectra as boundary conditions was applied. Experimentally obtained spectral emissivity and absorptivity were used to solve the heat balance equation to compute the net radiative cooling power and assess the cooling performance of the coating.

Outdoor temperature measurements were conducted under sunny conditions in Kunming, China. Measurements using aluminum-alloy and concrete substrates were performed on 15 July and 20 July 2026, respectively. The substrate dimensions were 50 × 50 × 2 mm^3^ for aluminum-alloy plates and 50 × 50 × 6 mm^3^ for concrete plates. All sprayed coatings had a dry-film thickness of 120 ± 10 μm. K-type thermocouples were affixed to the rear surfaces of the substrates using thermally conductive adhesive, with the junctions shielded from direct solar irradiation. The samples were thermally isolated from the ground using PE foam. Two parallel specimens were tested for each sample group, and the reported temperature data represent the mean values. The measurement uncertainty of the K-type thermocouples was ±0.5 °C.

CIELAB (L*, a*, b*) values were determined from diffuse-reflectance spectra measured using a PerkinElmer Lambda 950 UV–Vis–NIR spectrometer equipped with an integrating sphere. Measurements were performed using CIE standard illuminant D65 and the CIE 1931 2° standard observer under specular-component-included (SCI) geometry. BaSO_4_ reference calibration and dark-current correction were performed prior to measurements. For each coating, five measurements at different surface locations were averaged. The measurement uncertainties were ±0.3, ±0.2, and ±0.2 for L*, a*, and b*, respectively.

## 3. Results and Discussion

**Figure 1:**
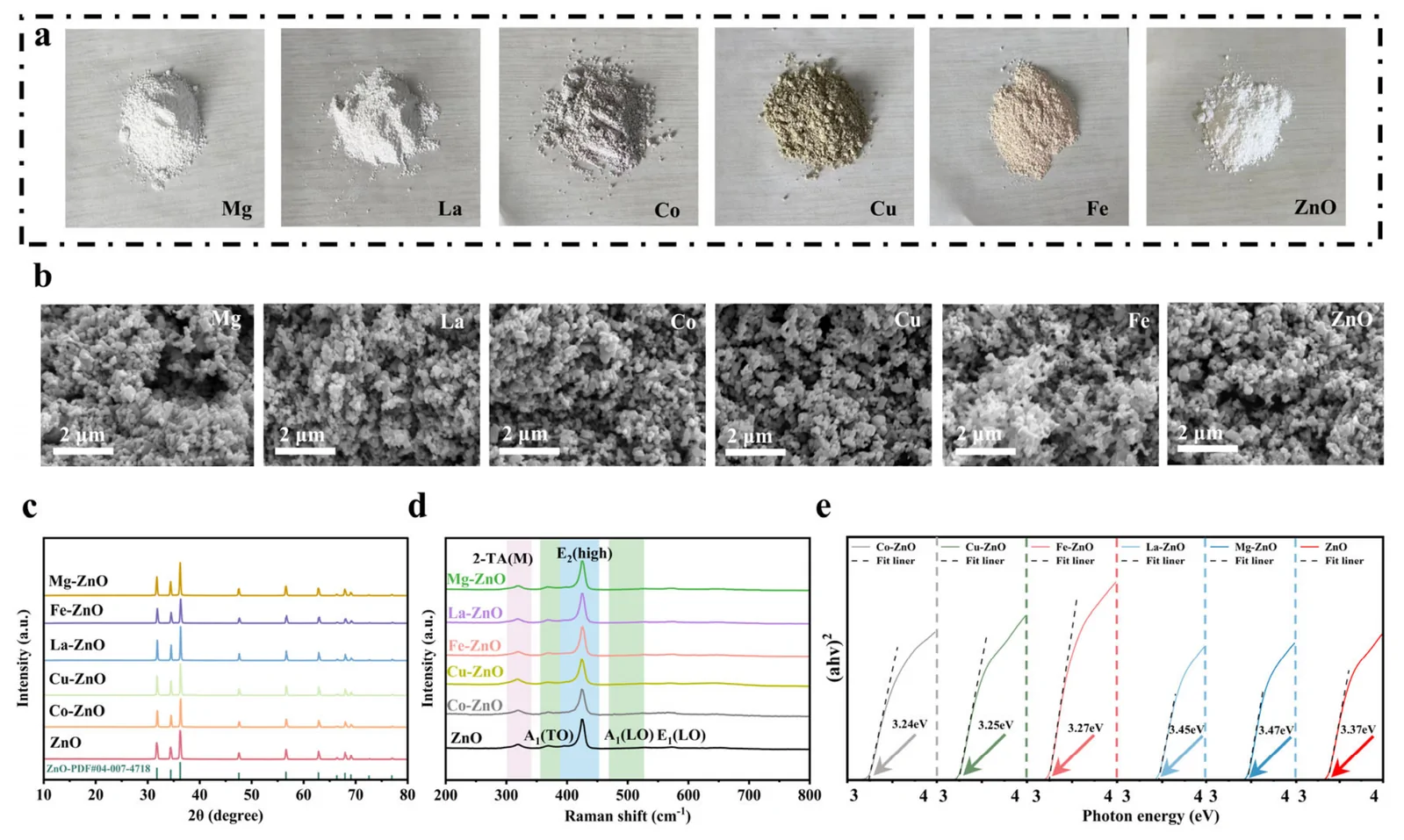
Physical characterizations on doped ZnO samples. (**a**) Digital photos. (**b**) SEM images. (**c**) XRD patterns. (**d**) Raman spectra. (**e**) Calculation about band gaps with UV–Vis spectra.

As expected, a low level (0.5 at%) of doping with Fe, Cu and Co enables the coloration of ZnO powders with pale buff (L* = 84.6, a* = 2.9, b* = 16.3), olive green (L* = 66.8, a* = −7.9, b* = 18.7), and pale gray (L* = 81.5, a* = −0.6, b* = 2.1) (Figure 1a) due to the existence of d-d transitions of these metal ions [35,36]. In addition, although the morphologies of the doped ZnO particles are changed due to the crystal reconstruction under annealing processes (Figure 1b) [37,38], all the ZnO-based powders present similar particle diameter distribution characters, indicating their similar solar diffraction behaviors. The influence of doping on the crystal structure of ZnO particles is further investigated by XRD and Raman measurements. As shown in Figure 1c, compared to the XRD signals of the pure hexagonal ZnO sample, the combination of none-impurity signals and unchanged ZnO diffraction patterns imply that the secondary metal ions should be fused into the crystal lattices of ZnO host. Additionally, benefiting from the low doping level of 0.5 at%, the unchanged Raman signals of all ZnO samples (Figure 1d) indicate that the doped metal ions do not change the crystal lattice vibrations [39,40], which indicates the almost unchanged MIR emission behaviors [41,42]. The EDS mappings (Figures S1–S5), acquired under long-exposure conditions, indicate that the metal elements are distributed within the ZnO particles. The band gaps of these ZnO-based samples were calculated to further analyze the influence of metal ion doping on the solar absorption behaviors. Based on the UV–Vis spectra, the band gap of the samples is calculated with Equation (1) [29]:

(1) $${\left[F\left(R\right)h\nu \right]}^{2}=A\left(h\nu -E_{g}\right),$$

where *F*(*R*) is the Kubelka–Munk function derived from the diffuse reflectance spectrum, *h* is Planck’s constant, *ν* is the photon frequency, *A* is a constant related to the transition probability, and *E_g_* is the optical band gap energy. As shown in Figure 1e, compared to the value of 3.37 eV for pure hexagonal ZnO, La- and Mg-doping methods lead to the increase of band gaps to 3.45 and 3.47 eV, which may be tentatively ascribed to the Burstein–Moss effect, band uplift induced by Mg^2+^ substitution alloying and the donor effect of La^3+^ dopants [43,44], though carrier-concentration measurements are needed for conclusive proof, while decreased band gaps of 3.27, 3.25 and 3.24 eV are observed for Fe-, Cu- and Co-doped ZnO samples due to the introduction of localized impurity states from transition-metal 3d orbitals and enhanced hybridization between Zn 3d–O 2p and TM 3d orbitals, generating intermediate energy levels that reduce electron transition energy [39,45]. The relatively small band gap differences indicate that these ZnO-based samples could present acceptable solar energy reflective capabilities [25,46].

**Figure 2:**
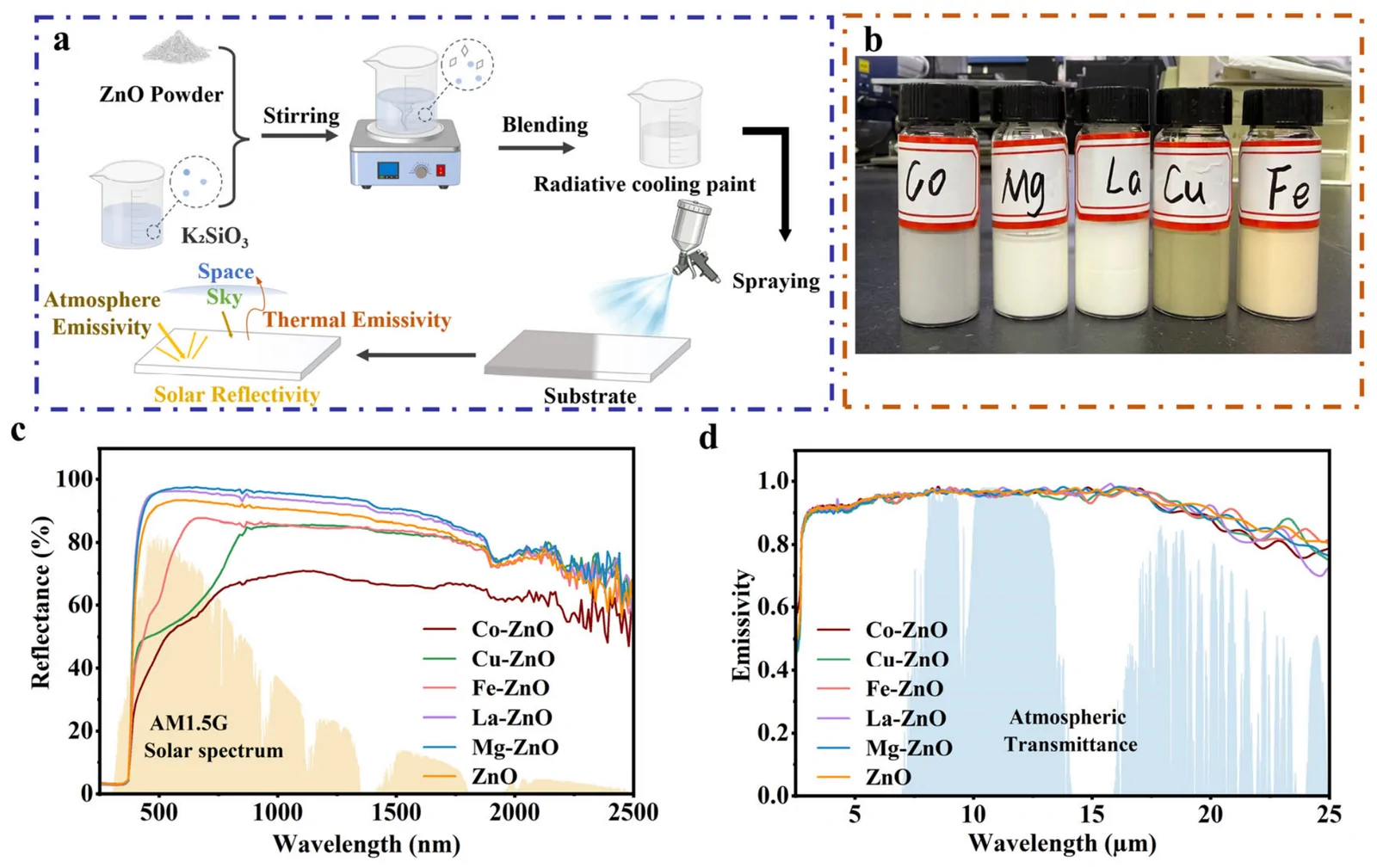
Fabrication and optical properties of ZnO-based paints. (**a**) Paint coating fabrication process. (**b**) Digital photos. (**c**,**d**) Solar reflective (250–2500 nm) and MIR (2.5–25 μm) emission spectra of pure and doped ZnO paint coatings.

Aqueous K_2_SiO_3_ solution is employed as a binder because of its high outdoor environmental stability, low cost, nonhuman toxicity, and strong attachment to various substrates, including concrete [47]. The stable paint formed with ZnO filler and K_2_SiO_3_ binder is formed by ball milling and sprayed on substrates with high-pressure air flow (Figure 2a,b). SEM images of the coatings are provided in Figure S6, showing homogeneous particle distributions and similar particle sizes with no obvious differences in surface morphology. After being treated with full conservation, the optical characters are detected. As shown in Figure 2c, due to the enlarged band gaps, the Mg and La doping methods lead to solar reflectance of 89.4% and 90.5%, compared to a value of 84.9% for the pure ZnO sample. In contrast, due to the narrowed band gaps of doped fillers, the Fe-, Cu- and Co-doped ZnO paint coatings show decreased solar reflectance of 75.7%, 65.6% and 57.4%, respectively. For the MIR emission behaviors, as reflected by the unchanged crystal structure demonstrated in Raman spectra, all the ZnO-based samples demonstrate high IR emissivity with values between 0.96 and 0.97 (Figure 2d). Such high IR emissivity indicates that all the prepared paint demonstrates high heat dissipation capabilities.

**Figure 3:**
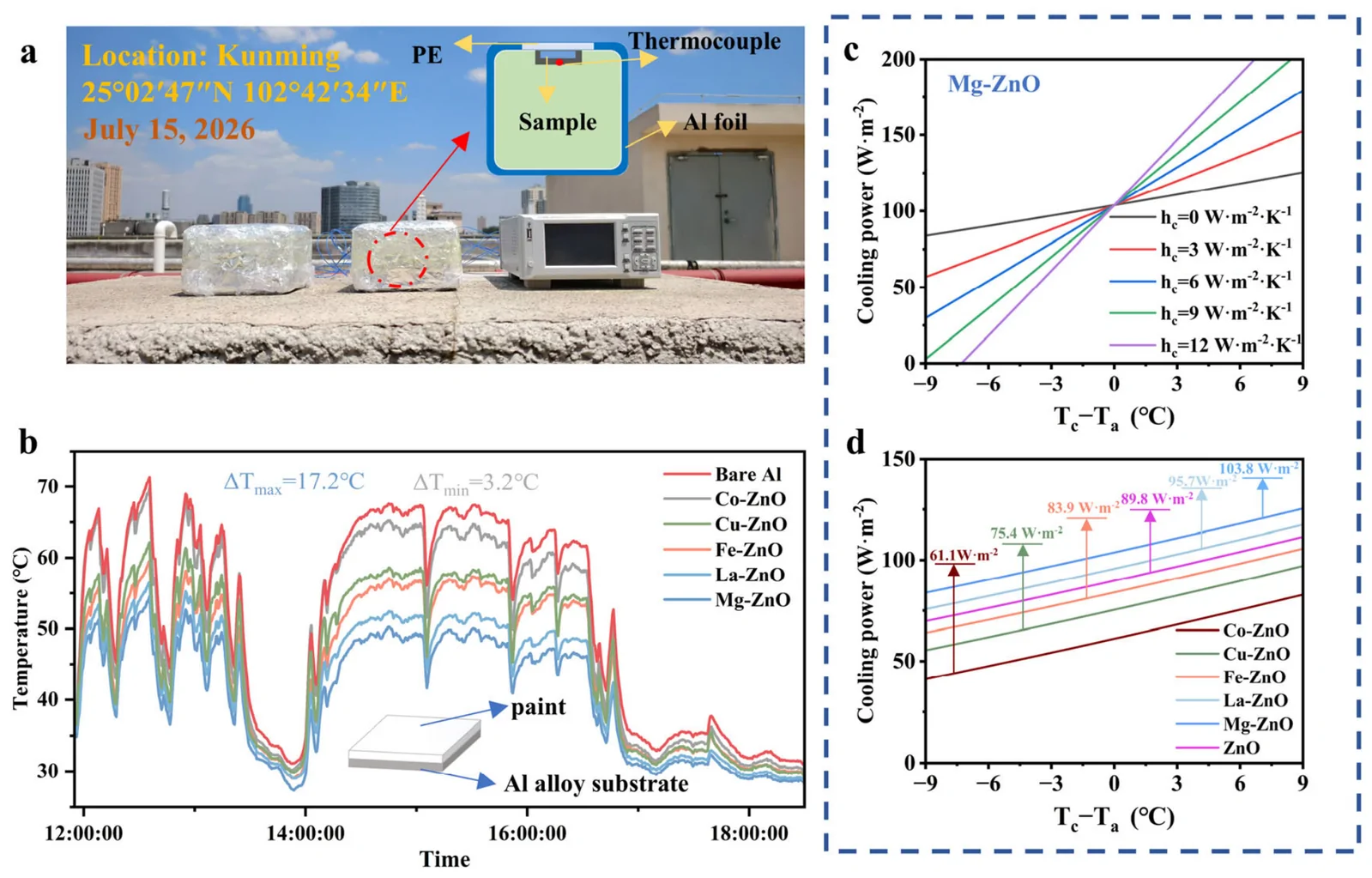
Theoretical characterizations on the cooling capabilities of doped ZnO-based paints. (**a**) Photograph and schematic of an outdoor cooling testing site in Kunming, China. (**b**) Comparisons of the daytime temperature of the pure and doped ZnO paint coatings on Al alloy substrates. (**c**) Calculated net cooling power during the daytime of Mg-doped ZnO paint coating. The variable h is a combined nonradiative heat coefficient. (**d**) Comparisons of the net cooling powers of the pure and doped ZnO paint coatings.

To more clearly demonstrate the cooling capabilities of the paints, the paints were sprayed on the surfaces of 6061 aluminum alloy plates and tested in outdoor chambers under a solar irradiance of 830 W m^−2^ during 12:00–18:00 without heat conduction and convection on 15 July 2026, in Kunming, China (Figure 3a). The comparisons of the daytime temperature vibrations are shown in Figure 3b. Under a sunny day with strong solar irradiation, all ZnO-based paint coatings demonstrate temperature decrease characteristics. Maximum temperature drop of 17.2 °C at noon with average daytime temperature differences of 9.6 °C are observed for the Mg-doped ZnO paint coating. For the Fe-, Cu- and Co-doped paints, although coloration decreased solar energy reflectance capabilities to some extent, they still demonstrate attractive temperature decrease capabilities. These three paint coatings show appealing temperature differences of 9.2, 7.3 and 3.2 °C at 3 p.m. with average temperature differences of 7.1, 5.4 and 1.7 °C, respectively. The Co-doped ZnO paint coating demonstrates the lowest temperature decrease performance, which can be attributed to its lowest solar reflectivity. The temperature-decreasing capability of the paint coating was further quantitatively evaluated by calculating the theoretical cooling power under a solar radiation of 600 W m^−2^ with an average environment temperature of 30 °C. Based on energy balance analysis, the net cooling power (P_net_) is calculated when the sample temperature (Ts) equals the ambient temperature (T_amb_). The nonradiative heat transfer coefficient (h = 0, 3, 6, 9, and 12 W m^−2^ K^−1^) on the net cooling performance was investigated (Figure 3c). As shown in Figure 3d, under optimal conditions, compared to pure ZnO paint (89.8 W m^−2^), the enlarged band gaps of the La- and Mg-doping methods result in improved cooling powers of 95.7 and 103.8 W m^−2^, respectively. As to the colored paints, although the decreased solar reflectance characters reduce cooling powers to some extent, the Fe-, Cu- and Co-doped ZnO paint coatings still demonstrate desirable cooling powers of 83.9, 75.4 and 61.1 W m^−2^, respectively. The detailed energy-balance formulation for calculating the net cooling power is described in Section S1 of the Supporting Information.

**Figure 4:**
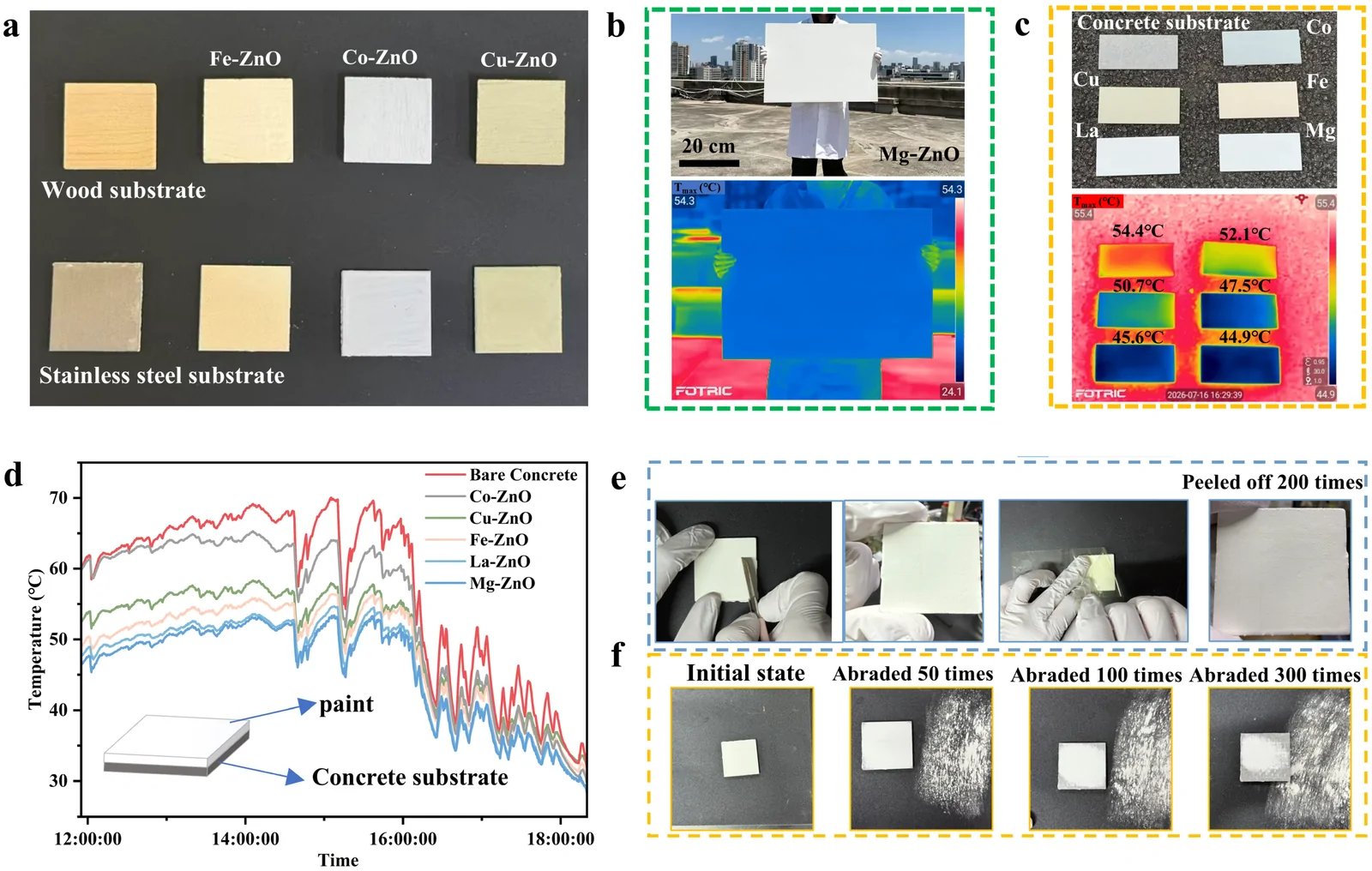
Physical characterizations on the cooling properties of the doped ZnO paints on building materials. (**a**) Photographs of ZnO paints on wood and steel substrates. (**b**) IR photos of the Mg-doped ZnO paint sprayed on wood substrate. (**c**) IR photos of the doped ZnO paints on concrete substrates. (**d**) Comparisons of the daytime temperature of the pure and doped ZnO paint coatings on concrete substrates. (**e**,**f**) Adhesion tests of Mg-doped ZnO paint coatings sprayed on concrete substrates in cross-hatch tape peeling and sandpaper abrasion peeling processes, respectively.

The paints were further sprayed on more typical building materials, including wood, steel and concrete, to demonstrate the practical applications. The concrete substrate is formed by being highly pressed with a thickness of 6 mm. As demonstrated in Figure 4a,b, the paints can be uniformly attached to wood and steel substrates, as well as demonstrating obvious temperature decrease capability. More notably, the paints can be coated on the concrete substrates and show obvious cooling characters (Figure 4c). Under direct sunlight radiation at noon, the concrete absorbs solar energy, leading its surface temperature to be quickly increased to 54.4 °C. While the doped ZnO paints, such as Mg- and Fe-doped ZnO fillers, benefited from their attractive cooling capabilities (103.8 and 83.9 W m^−2^), paint coatings make the surface temperatures decrease to 47.5 and 44.9 °C, respectively. Without eliminating the influence of heat convection from air flow, the temperatures on the bottom sides of these concrete samples were detected in an outdoor environment on 20 July 2026, in Kunming, China. When the temperature on the bottom side of the pure concrete plate is set as ambient temperature, as shown in Figure 4d, under solar radiation of 910 W m^−2^ during the period of 12:00–16:00, the average temperature decrease values are 7.5, 7.1, 4.6, 3.7 and 1.5 °C for the Mg-, La-, Fe-, Cu-, and Co-doped ZnO paints, respectively. Given the fact that concrete is the most widely employed wall material with low heat conductivity in practical buildings, the attractive temperature decrease phenomena on the concrete substrates confirm the promising application values of these developed paints. In addition, under an adhesion test of the cross-hatch tape process (Figure 4e), paint coating behaves with strong adhesion on the concrete substrate. While only part of the coating layer peels off when abrasive paper is used for intensive friction in the sandpaper abrasion test (Figure 4f). Such highly strong adhesion of the paint coating layer on the concrete substrate can be derived from the similar chemical characters between the aqueous K_2_SiO_3_ solution and the concrete [48,49,50]. Nevertheless, standardized durability testing remains necessary to assess the long-term outdoor performance of these coatings under realistic building conditions.

## 4. Conclusions

In conclusion, colored reflective paints with high cooling capabilities are realized by employing metal ion-doped ZnO fillers for building cooling and aesthetics. With an inorganic binder of aqueous K_2_SiO_3_ solution, the prepared paints can be tightly attached to various building materials, including wood, steel and concrete substrates. The Fe-, Cu- and Co-doping methods created pale buff (L* = 84.6, a* = 2.9, b* = 16.3), olive green (L* = 66.8, a* = −7.9, b* = 18.7), and pale gray (L* = 81.5, a* = −0.6, b* = 2.1) colors with high retained cooling powers of 83.9, 75.4 and 61.1 W/m^2^, respectively. Additionally, the colored coatings demonstrate promising cooling performance and good adhesion to concrete substrates under laboratory-scale conditions, while further standardized durability testing is needed to assess their long-term outdoor performance prior to practical building applications.

## Data Availability

The original contributions presented in the study are included in the article. Further inquiries can be directed to the corresponding authors.

## Supplementary Materials

The following supporting information can be downloaded at: https://www.mdpi.com/article/10.3390/nano16181155/s1. Table S1: Fitting parameters and uncertainties of the optical band gaps determined from Tauc plots; Figure S1. SEM image and EDS mappings (Cu, O, Zn) of Cu-doped ZnO; Figure S2. SEM image and EDS mappings (La, O, Zn) of La-doped ZnO; Figure S3. SEM image and EDS mappings (Fe, O, Zn) of Fe-doped ZnO; Figure S4. SEM image and EDS mappings (Mg, O, Zn) of Mg-doped ZnO; Figure S5. SEM image and EDS mappings (Co, O, Zn) of Co-doped ZnO; Figure S6. SEM images of Co-, Cu-, Fe-, Mg-, and La-doped ZnO coatings and the ZnO coating; Section S1: Fundamentals of passive daytime radiative cooling.
