# Supplementary material for: Colored Reflective Paints with Doped ZnO Fillers for High Cooling Capabilities
Source: Nanomaterials (Basel). 2026 Sep 15;16(18):1155. doi: 10.3390/nano16181155 (PMC13610355; doi:10.3390/nano16181155)
Supplement: Supplementary file 1 [file nanomaterials-16-01155-s001.zip › nanomaterials-4544662-supplementary.pdf]

Table S1: Fitting parameters and uncertainties of the optical band gaps determined from Tauc plots

Sample	Fitting range (eV)	Eg (eV)	R^2	Fitting uncertainty (eV)
Co-ZnO	3.179–3.351	3.24	0.9856	±0.018
Cu-ZnO	3.263–3.444	3.25	0.9915	±0.012
Fe-ZnO	3.263–3.444	3.27	0.9921	±0.011
ZnO	3.380–3.561	3.37	0.9956	±0.008
La-ZnO	3.387–3.558	3.45	0.9970	±0.010
Mg-ZnO	3.395–3.567	3.47	0.9980	±0.014

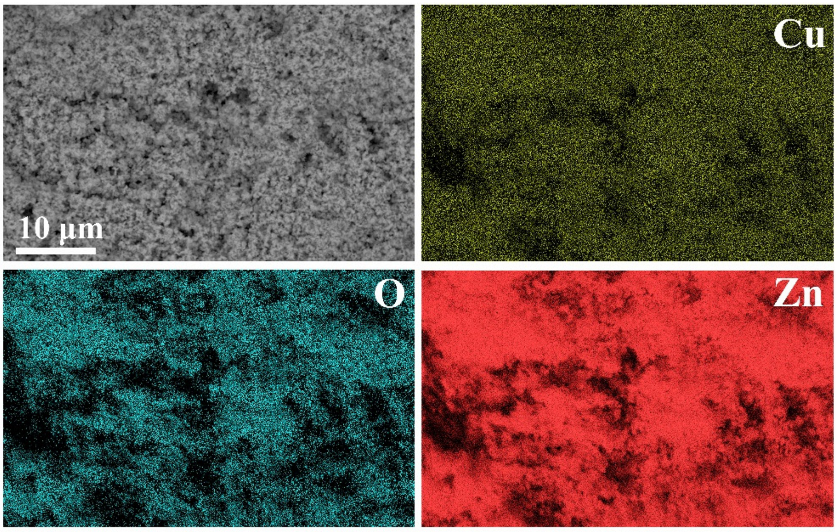

Figure S1. SEM image and EDS mappings (Cu, O, Zn) of Cu-doped ZnO

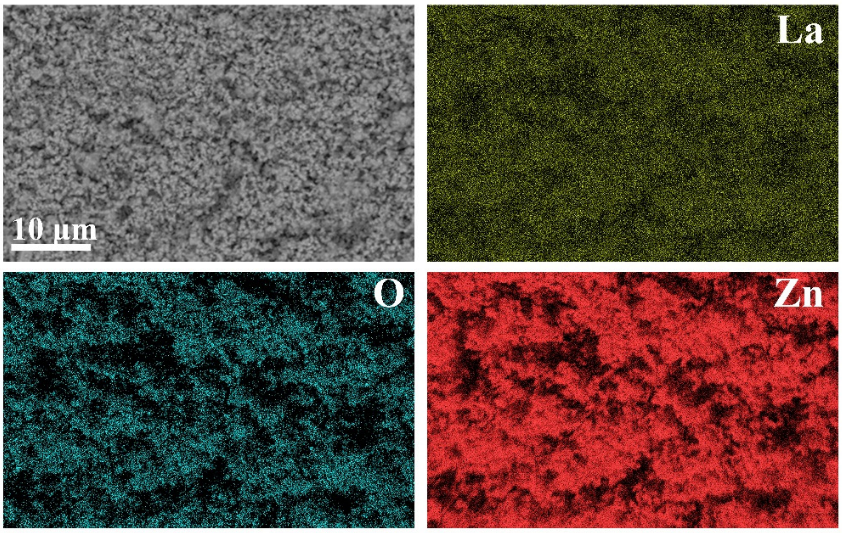

Figure S2. SEM image and EDS mappings (La, O, Zn) of La-doped ZnO

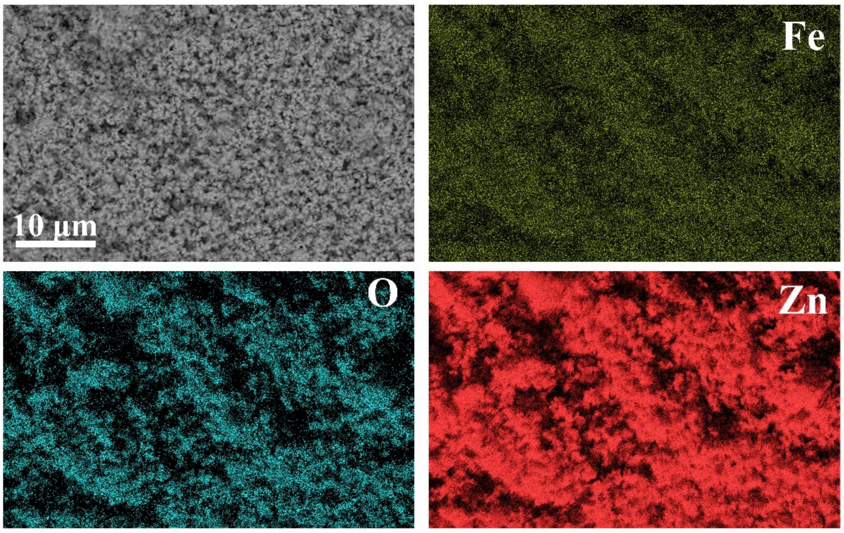

Figure S3. SEM image and EDS mappings (Fe, O, Zn) of Fe-doped ZnO

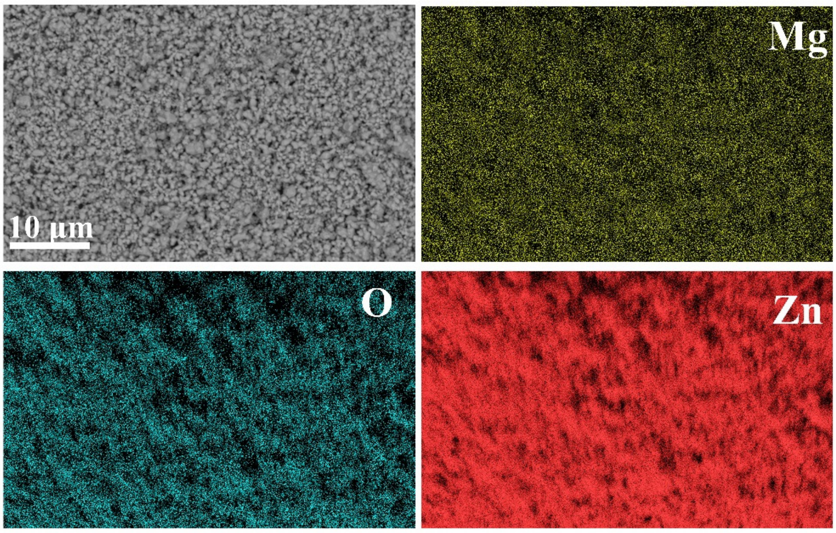

Figure S4. SEM image and EDS mappings (Mg, O, Zn) of Mg-doped ZnO

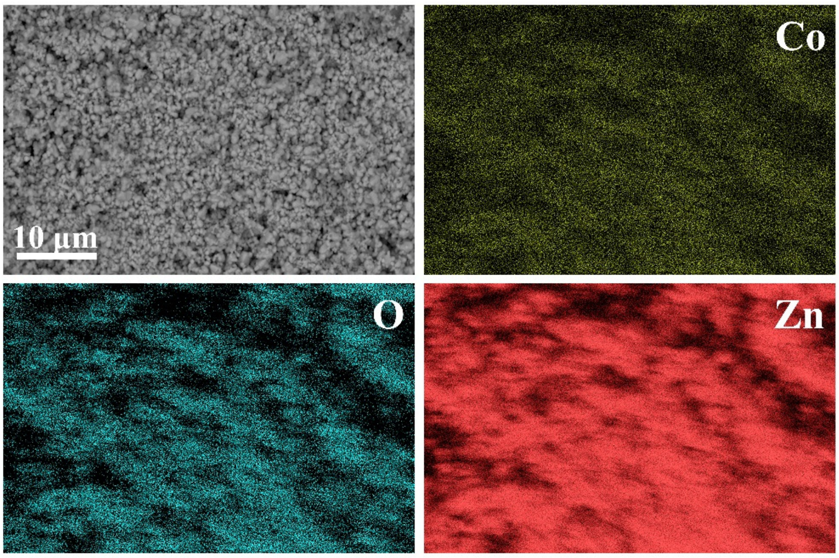

Figure S5. SEM image and EDS mappings (Co, O, Zn) of Co-doped ZnO

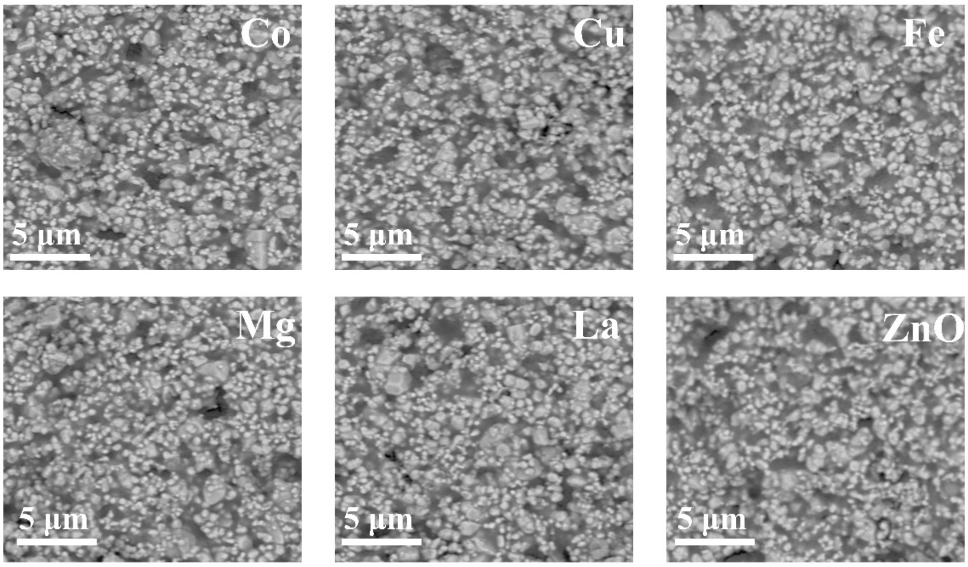

Figure S6. SEM images of Co-, Cu-, Fe-, Mg-, and La-doped ZnO coatings and the ZnO coating.

Section S1: Fundamentals of passive daytime radiative cooling

The net radiative cooling power of the material can be expressed as:

$$P_{net(T)} = P_{rad}(T) - P_{atm}(T_{amb}) - P_{solar} - P_{cond+conv}(S1)$$

where $P_{rad}(T)$ is the thermal radiative power emitted by the film itself, $P_{atm}(T_{amb})$ is the thermal radiative power of the ambient atmosphere absorbed by the film, P_{solar} is the solar radiation power absorbed by the material, and $P_{cond+conv}$ is the total heat loss due to conduction and convection.

The thermal radiative power of the film is calculated as:

$$P_{rad}(T) = 2\pi \int_0^{\frac{\pi}{2}} d\theta\, sin\theta \int_0^{\infty} d\lambda\, I_{BB}(T, \lambda)\varepsilon(\lambda, \theta)(S2)$$

The atmospheric thermal radiative power absorbed by the film is calculated as:

$$P_{atm}(T_{amb}) = \int d\Omega\, cos\theta \int_0^{\infty} d\lambda\, Ibb(Tamb, \lambda)\varepsilon(\lambda, \theta)\varepsilon atm(\lambda, \theta)(S3)$$

The angular-dependent atmospheric emissivity is calculated as:

$$\varepsilon_{atm}(\lambda, \theta) = 1 - \tau(\lambda)^{1/cos\theta}(S4)$$

where $\tau(\lambda)$ is the atmospheric transmittance in the zenith direction.

The solar radiation power absorbed by the film is calculated as:

$$P_{solar} = \int_0^{\infty} d\lambda\, \varepsilon(\lambda, \theta solar) I_{AM1.5}(\lambda)(S5)$$

The non-radiative heat loss is calculated as:

$$P_{cond+conv}((T, T_{amb}) = hc(T_{amb} - T)(S6)$$

where $h_c = h_{cond} + h_{conv}$ is the total heat transfer coefficient.
